# Our Premium Offer for 1886

**Published:** 1885-12-20

**Authors:** 


OUR PREMIUM OFFER FOR 1886.—To any one sending us two new subscribers, with $4 remitted, we will send a copy of "Hall's Problem of Human Life," a large $2 book—a work of extraordinary ability and interest, and now attracting great attention throughout the civilized world. It would constitute a splendid Christmas or New Year's gift to a friend.

